# Most surgeons still prefer to reduce overriding distal radius fractures in children

**DOI:** 10.1080/17453674.2020.1854502

**Published:** 2020-12-10

**Authors:** Topi Laaksonen, Jani Puhakka, Jussi Kosola, Antti Stenroos, Matti Ahonen, Yrjänä Nietosvaara

**Affiliations:** a Department of Pediatric Orthopedics and Traumatology, Helsinki New Children’s Hospital;; b Department of Orthopedics and Traumatology, Töölö Hospital, Helsinki University Hospital, Finland

## Abstract

Background and purpose — Traditionally, overriding distal radius fractures in children have been reduced and immobilized with a cast or treated with percutaneous pin fixation. There is recent evidence that these fractures heal well if immobilized in the bayonet position without reduction. We evaluated the present treatment of these fractures.

Methods — A questionnaire including AP and lateral radiographs of overriding distal radius fractures in 3 pre-pubertal children was answered by 213 surgeons from 28 countries. The surgeons were asked to choose their preferred method of treatment (no reduction, reduction, reduction and osteosynthesis), type and length of cast immobilization, and the number of clinical and radiographic follow-ups.

Results — Of the 213 participating surgeons, 176 (83%) would have reduced all 3 presented fractures, whereas 4 (2%) would have treated all 3 children with cast immobilization without reduction. Most reductions (77%) would have been done under general anesthesia. Over half (54%) of the surgeons who preferred anesthesia would have fixed (pins 99%, plate 1%) the fractures. An above-elbow splint or circular cast was chosen in 84% of responses, and the most popular (44%) length of immobilization was 4 weeks. Surgeons from the Nordic countries were more eager to fix the fractures (54% vs. 31%, p < 0.001) and preferred shorter immobilization and follow-up times and less frequent clinical and radiological follow-ups compared with their colleagues from the USA.

Interpretation — Most of the participating surgeons prefer to reduce overriding distal radius fractures in children under anesthesia. There is substantial lack of agreement on the indications for osteosynthesis, type of cast, length of immobilization, and follow-up protocol.

Most authors recommend reduction of overriding distal radius fractures in children (McLauchlan et al. 2002, Miller et al. 2005, Zamzam and Khoshhal 2005, Wendling-Keim et al. 2014). Routine percutaneous pin fixation has also been advocated because these fractures have a high risk of re-displacement after reduction (McLauchlan et al. 2002, Zamzam and Khoshhal 2005, Alemdaroğlu et al. 2008, Hang et al. 2011). On the other hand, Do et al. (2003) and Crawford et al. (2012) have reported good results after cast immobilization without fracture reduction regardless of fracture displacement.

We assessed current treatment preferences in overriding distal radius fractures in pre-pubertal children. The secondary aim was to record the proposed types of cast, the length of immobilization, and the number of clinical and radiological follow-ups from different institutions.

## ^Methods^

This survey was designed to assess current opinions and practices in the treatment of overriding distal metaphyseal fractures of the radius in less than 10-year-old children. Participation was voluntary, and no compensation was given. The SurveyMonkey™ (San Mateo, CA, USA) website served as a platform to collect and store responses.

**Figure 1.**
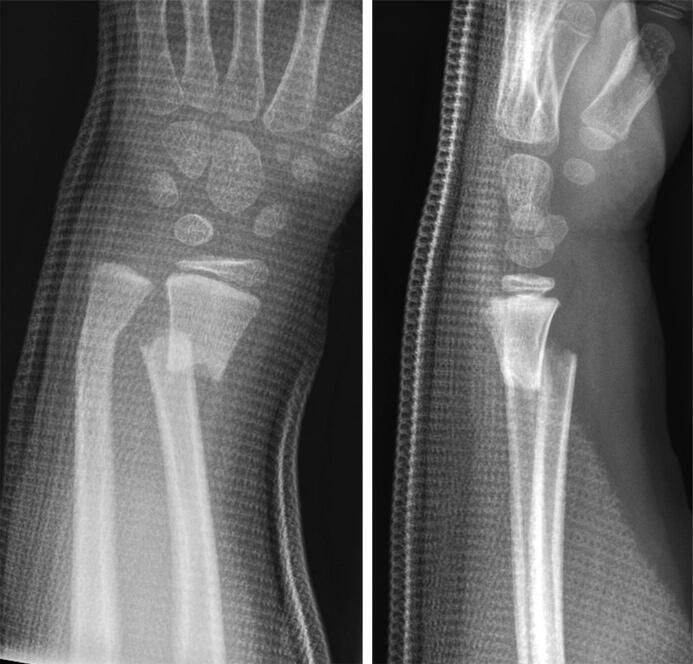
Case 1: a 5-year-old girl who fell of a swing and sustained a completely displaced distal radius fracture with shortening and a non-displaced but slightly angulated fracture of the distal ulna.

**Figure 2.**
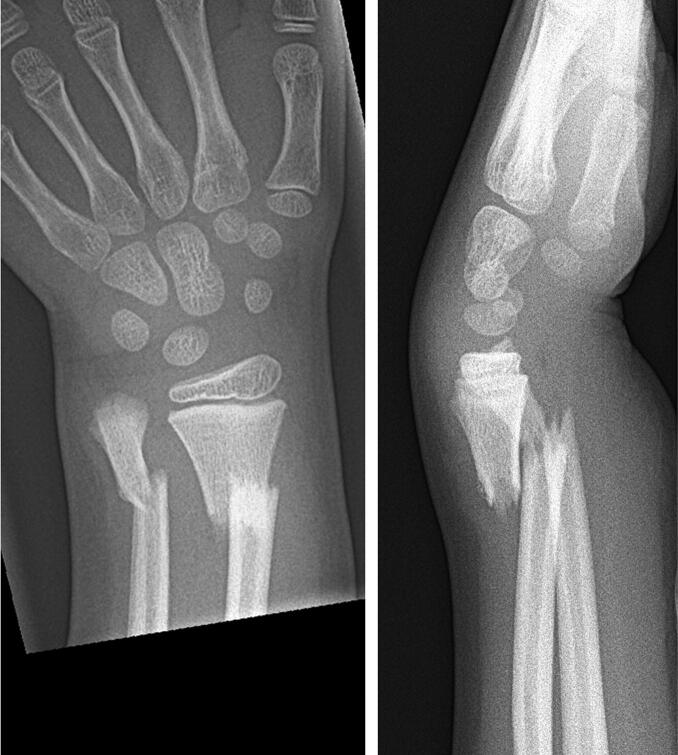
Case 2: a 7-year-old boy who fell off a climbing frame and sustained a completely displaced distal radius fracture with shortening and a subtotally displaced fracture of the distal ulna with some angulation.

**Figure 3.**
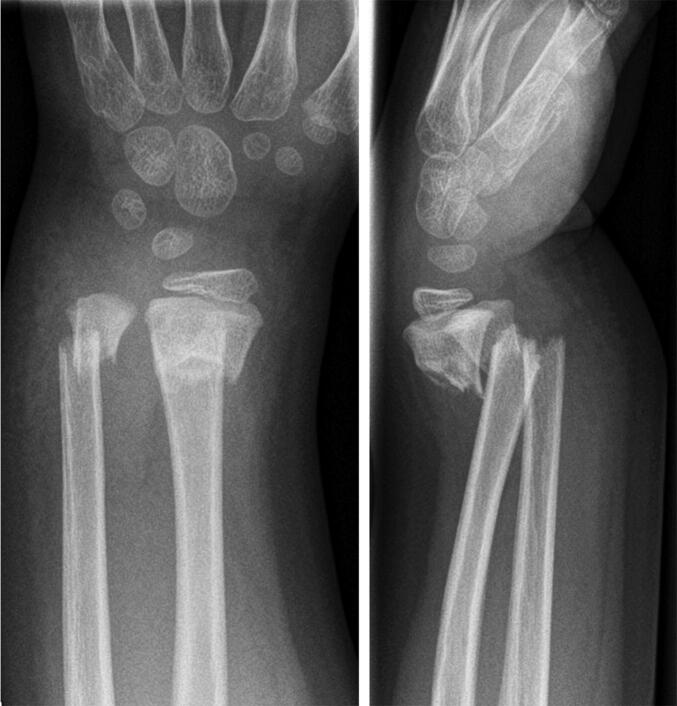
Case 3: a 5-year-old boy who fell off a climbing frame sustaining completely displaced and shortened fractures of both radius and ulna with angulation.

3 otherwise healthy, aged in accordance with study criterion, children’s overriding (complete displacement and shortening) distal metaphyseal radius fractures (Figures 1–3) with different types of distal ulnar fractures were presented. Age, mechanism of injury, and both anteroposterior (AP) and lateral radiographs were shown. The participants were asked to choose their preferred method of treatment, type and length of cast immobilization, and the number of clinical and radiographic follow-ups (Table 1). Results were analyzed based on treatment method in 3 groups: (1) no reduction, (2) reduction, and (3) reduction and osteosynthesis (Table 2).

**Table 1.** Survey questions and reply alternatives

Method of treatment	Cast immobilization without reduction
	Alignment adjustment during casting
	Reduction in local anesthesia
	Reduction under anesthesia
	Percutaneous pin fixation
	Open reduction and pin fixation
	Plate fixation
Type of cast	None
	Dorsal forearm splint
	Dorsal and volar forearm splint
	Above-elbow dorsal splint
	Above-elbow dorsal and volar forearm splint
	Circular forearm cast
	Circular above-elbow cast
Weeks of immobilization	None, 1, 2, 3, 4, 5, 6 or >6
Number of	
radiological follow-ups	None, 1, 2, 3, 4, 5, 6 or >6
clinical follow-ups	None, 1, 2, 3, 4, 5, 6 or >6

**Table 2.** Distribution of all 639 responses given by the 213 surgeons participating in the survey

Case no.	1	2	3	Total
No reduction				
Cast immobilization only	22	10	20	52
Alignment adjustment during casting	17	7	19	43
Total	39	17	39	95
Reduction				
with local anesthesia	16	6	28	50
under anesthesia	93	93	42	228
Total	109	99	70	278
Reduction and osteosynthesis				
Percutaneous pin fixation	58	86	49	193
Open reduction and pin fixation	6	10	55	71
Plate fixation	1	1	0	2
Total	65	97	104	266

Case 1: a non-displaced but slightly angulated fracture of the distal ulna (Figure 1).

Case 2: a sub-totally displaced fracture of the distal ulna with some angulation (Figure 2).

Case 3: a completely displaced and shortened fracture of the ulna with angulation (Figure 3).

A query was sent to heads of several pediatric orthopedic departments in Europe (n = 25) and in North America (n = 9) to be circulated to attending surgeons and registrars treating children’s fractures. An additional 48 respondents who claimed to treat children’s fracture in their practice were recruited from the European Pediatric Orthopedic Society (EPOS) meeting in Tel Aviv 2019. Respondents’ experience in the field of pediatric orthopedics and their country of practice were registered anonymously. The survey was completed by 213 surgeons from 21 countries (Table 3). Among the respondents, 110 (52%) had more than 5 years of experience in pediatric orthopedics. There were 94 respondents from the Nordic countries and 50 from the USA, with a similar profile of pediatric orthopedic experience. The response rate concerning the e-mailed queries is unknown, but only 1 of 49 surgeons asked to participate in the survey at the EPOS meeting declined.

**Table 3.** Pediatric orthopedic experience and country of the 213 participants

Factor	n (%)
Experience	
< 1 year	47 (22)
1–5 years	56 (26)
6–10 years	34 (16)
> 10 years	76 (36)
Country	
Finland	73 (34)
USA	50 (24)
Sweden	14 (7)
Australia	9 (4)
Austria	7 (3)
UK	7 (3)
Estonia	6 (3)
Norway	6 (3)
Switzerland	5 (2)
France	5 (2)
Russia	5 (2)
Germany	4 (2)
Israel	4 (2)
Other **^a^**	18 (9)

**^a^** Fewer than 3 respondents from Armenia, China, Denmark, Japan, Northern Ireland, Mexico, Portugal, and Singapore.

### ^Statistics^

The response distribution for each individual question was analyzed, and the agreement between surgeons was determined using Cronbach’s α with a cutoff value of 0.8. Values below 0.5 are considered unacceptable (Cortina 1993). Binary logistic regression analysis was performed to determine which parameter (ulnar fracture type, surgeon’s experience in pediatric orthopedics, country of origin) was of greatest and most independent significance for the prediction of osteosynthesis. Statistical analysis among countries was done only between Nordic countries (Finland, Denmark, Norway, and Sweden) and the USA due to the small number of respondents from other countries. Demographic data were explored using a chi-square test and Pearson’s correlation with a p-value of < 0.05 to define statistical significance. All analyses were performed using SPSS for Windows (IBM Statistics for Windows, Version 22.0, released 2013, IBM Corp, Armonk, NY, USA).

### ^Ethics, funding, and potential conflicts of interest^

The institutional research ethics committee approved the study, and the principle of implied consent was applied, thus formal consent was not required. Study and consent details were clearly communicated before respondents began the questionnaire. The study was supported by Finska Läkaresällskapet (the Medical Society of Finland). There were no potential conflicts of interest. 

## ^Results^

Of the 213 participating surgeons, 176 (83%) would have reduced all the fractures with or without fixation, whereas 4 respondents (2%) would have treated all 3 children with cast immobilization without reduction. General anesthesia would have been performed in 77% of the fracture reductions. Surgeons who would have liked to stabilize these fractures opted for pins in 99% of cases. There was no difference between respondents from the Nordic countries and those from the USA concerning the rate of reduction (87% vs. 89%), but surgeons from the Nordic countries were more eager to use pins to fix the fractures compared with their American colleagues (54% vs. 31%, p < 0.001).

The majority (84%) of all surgeons chose an above-elbow splint or cast to immobilize the presented fractures. Splints were more popular than circular casts, especially for surgeons who advocated reduction and percutaneous pin fixation (61% vs. 39%, p < 0.001). The responses concerning the length of immobilization varied widely, with the following distribution: no immobilization 0.8%, 2 weeks 1.4%, 3 weeks 13%, 4 weeks 44%, 5 weeks 14%, 6 weeks 26%, and > 6 weeks 0.8%. The median length of cast immobilization chosen by the Nordic surgeons was 4 weeks, whereas the American surgeons preferred 6 weeks.

There was also a wide variation in the preferred number of outpatient visits and follow-up radiographs. The median number of follow-up visits suggested by Nordic surgeons was 2 compared with the 3 recommended by surgeons from the USA (p = 0.003). Follow-up of longer than 2 months was suggested by 16% of the Nordic respondents and by 32% of the Americans.

Table 2 presents the distribution of answers in each patient case and the lack of agreement on the method of treatment (α = 0.12). Overall, participants showed internal consistency in the method of treatment (α = 0.86). There was no correlation with the type of ulnar fracture, the surgeon’s pediatric orthopedic experience, the preferred method of treatment, type and length of immobilization, or with the number of clinical and radiographic follow-ups that were suggested (p > 0.1). When all variables were analyzed by binary logistic regression analysis, the only attribute that correlated positively with pin fixation was Finland as the surgeon’s country of practice (OR 5.3, 95% CI 1.5–19). Conversely, the type of ulnar fracture (Case 1 – no displacement and no shortening) (OR 2.1, CI 1.1–3.7) and the length (> 10 years) of respondents’ pediatric orthopedic experience (OR 2.1, CI 1.3–3.2) correlated positively with nonoperative treatment. 

## ^Discussion^

**Figure 4.**
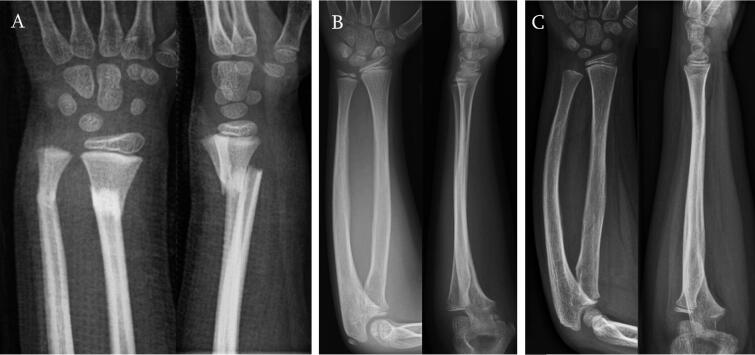
A–C: all 3 patients’ fractures were immobilized in bayonet position (A) without reduction with synthetic dorsal above-elbow and volar below-elbow splints applied in finger-trap traction without anesthesia in the emergency department. Splints were removed at 4 weeks. Radiographs at 3.5 years from the injury in case 1 (B) and case 3 (C). Parents of Case 2 (A) did not want any follow-up radiographs taken, because their son had made full functional recovery and he had no pain.

Do et al. (2003) and Crawford et al. (2012) have reported that overriding distal radius fractures in children can be treated by letting the fractures unite in the bayonet position in a cast, and uneventful remodeling will follow. According to our survey, a significant change in the traditional thinking that these fractures should be reduced has not taken place. The 3 fractures presented in the survey were healed in a bayonet position by 4 weeks (Figure 4) and were radiologically completely remodeled in 1 year (Figure 4). Full function was evident by 6 months.

A recent Cochrane analysis (Handoll et al. 2018) outlines the need for high-quality studies on whether cast immobilization has better results with no formal reduction or with closed reduction and percutaneous pin fixation of distal displaced forearm fractures in children. AO Surgery Reference Guidelines for treatment of displaced distal metaphyseal forearm fractures do not give an upper limit for angulation or shortening in children aged under 10 years. In other words, AO Surgery Reference Guidelines, used worldwide, do not give clear recommendations for children under 10 years of age. Could this be one of the reasons why most surgeons still reduce these fractures in all age groups?

In Bernthal et al. (2015) an internet-based survey on the management of pediatric distal radius fractures reveals that fewer than 10% of American surgeons recommend cast immobilization without reduction of overriding fractures as the primary treatment. Interestingly, the overriding fracture position was accepted by approximately half of the pediatric orthopedic surgeons at the 1-week follow-up. Several previous studies have recommended percutaneous pin fixation to avoid redisplacement (Gibbons et al. 1994, McLauchlan et al. 2002, Miller et al. 2005, Colaris et al. 2013). Our study results were similar to those of Bernthal et al. (2015): only a minority of surgeons would leave overriding distal radius fractures in children unreduced. Percutaneous pin fixation appears to be especially popular in the Nordic countries for no evident reason.

Above-elbow casts do not seem to retain alignment of distal forearm fractures in children any better than below-elbow casts, according to earlier reports (Bohm et al. 2006, Paneru et al. 2010). Nevertheless, most surgeons who participated in this survey preferred an above-elbow cast. We could not find any evidence in the literature on the superiority of either circular casts or splints in immobilizing distal forearm fractures in children, which aligns with the results of our study, with a relatively even distribution of circular casts and splints. Nordic surgeons seem to prefer splints in contrast to American surgeons, who favor circular casts.

There are no clear guidelines on the length of cast immobilization in children’s distal forearm fractures, which has varied from 4 to 6 weeks in previous studies (McLauchlan et al. 2002, Miller et al. 2005, Bohm et al. 2006, Paneru et al. 2010, Crawford et al. 2012, Colaris et al. 2013) In our study, 84% of responses fell into the 4–6-week category. Again, there was regional variability concerning the length of cast immobilization, as Nordic surgeons would generally remove casts at 4 weeks, 2 weeks earlier than their American colleagues.

Rockwood and Wilkins (Flynn et al. 2015) recommend repeated follow-ups weekly for the first 3 weeks to monitor alignment of distal forearm fractures in children, but they give no recommendations concerning the length of the follow-up. In addition, the guidelines from the recent Cochrane review (Handoll et al. 2018) are no better. Malunited distal forearm fractures in children are completely remodeled within 3 to 12 months with few exceptions (Do et al. 2003, Crawford et al. 2012). Therefore, routine radiographic controls and long-term follow-up seem unnecessary. Most respondents would have nevertheless arranged at least 2 or 3 clinical and radiographic follow-up examinations, presumably at least partially to monitor fracture alignment. Conversely, more than 80% of the participating surgeons would have discontinued follow-ups of their patients by 3 months. The number of suggested outpatient appointments and the length of the follow-up appear to be shorter in Nordic countries than in the USA, which could be partially explained by the higher rate of pin fixation in the Nordic countries. In the USA, longer and more frequent clinical and radiographic follow-up may represent defensive medicine regarding malpractice litigation.

The results of this study should be interpreted with caution because the respondents comprise only a small fraction of all surgeons treating pediatric fractures. Second, treatment decisions in this survey were based on radiographs and a short patient history. Actual bedside decisions could be different. Third, according to responses from some North American respondents, closed reduction with conscious sedation in the emergency room is a common method of treatment, which was not included as an option. Fourth, we did not present an overriding distal radius fracture with an intact ulna, which might have propelled more surgeons to choose the options that did not involve formal reduction of the fractures.

### ^Conclusion^

Based on our survey, the most common treatment method of overriding distal radius fractures in ` 10-year-old children is reduction under anesthesia and immobilization with an above-elbow cast. Percutaneous pin fixation is popular in the Nordic countries. Very few surgeons would treat these fractures without reduction. There is no consensus regarding the type of cast, the length of immobilization, or the number of follow-up examinations.

The reports of Do et al. (2003) and Crawford et al. (2012) have thus not led to a change in treatment praxis. We have therefore started a non-inferiority randomized controlled treatment trial registered in Clinical Trials (Casting in finger trap traction without reduction and percutaneous pin fixation of dorsally displaced, overriding distal forearm fractures in children under 11 years old, ClinicalTrials.gov Identifier: NCT04323410). 
